# A Long Journey Ahead: Long Non-coding RNAs in Bacterial Infections

**DOI:** 10.3389/fcimb.2017.00095

**Published:** 2017-03-28

**Authors:** Jennifer zur Bruegge, Ralf Einspanier, Soroush Sharbati

**Affiliations:** Department of Veterinary Medicine, Institute of Veterinary Biochemistry, Freie Universität BerlinBerlin, Germany

**Keywords:** long non-coding RNAs (lncRNAs), bacterial infection, epigenetic regulation of gene expression, host-pathogen interaction, immunity

## Abstract

Bacterial pathogens have coevolved with their hosts and acquired strategies to circumvent defense mechanisms of host cells. It was shown that bacteria interfere with the expression of mammalian microRNAs to modify immune signaling, autophagy, or the apoptotic machinery. Recently, a new class of regulatory RNAs, long non-coding RNAs (lncRNAs), was reported to have a pivotal role in the regulation of eukaryotic gene expression. A growing body of literature reports on specific involvement of lncRNAs in the host cell response toward bacterial infections. This mini review summarizes recent data that focuses on lncRNA function in host cells during bacterial infection and provides a perspective where future research in this regard may be going.

## Introduction

Interkingdom communication between hosts and microbes is one of the propelling aspects in mammalian evolution. Mucosal surfaces provide an extensive area, which is in direct contact with bacteria, fungi and viruses. Bilateral communication of the mucosal tissue with microbiota is essential for colonization, homeostasis, and development. On the other hand, bacterial pathogens have coevolved with their hosts and have acquired mechanisms to defeat mucosal barrier, invade and hijack phagocytes, and interfere with phagosome biogenesis or apoptotic pathways to manifest infection (Pieters, 2008; Rocco and Irani, 2011; Sharbati et al., 2011). Many bacterial pathogens such as *mycobacteria, listeriae*, and *salmonellae* can invade host cells and replicate inside.

Intracellular bacterial pathogens have been shown to interfere with expression of small non-coding, regulatory RNAs like microRNAs (miRNA) affecting host cell response signaling pathways such as apoptosis and autophagy (Rodriguez et al., 2007; Schulte et al., 2011; Sharbati et al., 2011, 2012; Hoeke et al., 2013; Pawar et al., 2016b; zur Bruegge et al., 2016). While the role of miRNAs in bacterial infections has been extensively studied and excellently reviewed in previous years (Eulalio et al., 2012; Das et al., 2016; Kim et al., 2017), we are beginning to realize the pivotal role of another class of regulatory RNA molecules, which are collectively referred to as long non-coding RNAs (lncRNAs). Next-Generation RNA sequencing (RNAseq) studies have examined that the majority of the mammalian genome is transcribed but very little of it has the ability to encode for proteins (Birney et al., 2007). lncRNAs are distinguished from other non-coding RNAs primarily based on their size of larger than 200 nt. Their numbers are estimated to reach those of protein coding genes but they are generally shorter, have fewer exons and possess low evolutionary conservation (Bertone et al., 2004; Heward and Lindsay, 2014). Furthermore, they have lower level of cellular concentration than protein-coding transcripts but have a higher degree of tissue specificity (Pang et al., 2006; Marques and Ponting, 2009). lncRNAs are frequently localized in the nucleus functioning both in *cis* (at the site of their transcription) and in *trans* (at the sites on other chromosomes), which points to potential functions as interfaces with the epigenetic machinery, chromatin organization, and regulation of gene expression. lncRNAs function e.g., as protein scaffolds, activators, or inhibitors of transcription, antisense RNA or miRNA sponges, respectively (Cech and Steitz, 2014; Rinn, 2014). The latter has been reported recently as a novel mode of action of lncRNAs, where they act as competing endogenous RNA (ceRNA). This suggests the existence of a network of lncRNAs, miRNAs, and mRNAs crosstalking based on mutual miRNA response elements (MRE; Salmena et al., 2011; Hanisch et al., 2017). There are many subclasses of lncRNAs, classified e.g., based on their length and location such as long intergenic RNAs (lincRNAs) or based on their association with annotated protein coding genes such as natural antisense transcripts (NATs; St Laurent et al., 2015). A growing body of literature reports on specific lncRNA involvement in host cell response to bacterial infections. This mini review is intended to summarize very recent data on lncRNA function in bacterial infections. We aim at combining these pioneering data with relevant cellular signaling pathways that are manipulated by bacterial pathogens to provide a perspective where further research in this regard may be going.

## Exploring lncRNA function through the analysis of neighboring coding genes

The fact that lncRNAs are prominently involved in the response of different host cells to various bacterial agents such as *Mycobacterium* (*M*.) spp., *Salmonella* (*S*.) Typhimurium, *Escherichia coli, Listeria* (*L*.) *monocytogenes, Helicobacter pylori*, and *Campylobacter* (*C*.) *concisus* as well as TLR ligands has been recently demonstrated in several studies but the functions of these lncRNAs remain to be elucidated (Ilott et al., 2014; Yi et al., 2014; Yang et al., 2015; Zhu et al., 2015; Yang R. et al., 2016; Westermann et al., 2016).

lncRNAs have been reported to regulate the expression of neighboring protein-coding genes through a locus control process (Wang and Chang, 2011; Wang et al., 2011). Therefore, functions may be speculated from their locus and adjacent protein coding genes. According to this assumption, genes related to immune functions are most likely influenced by lncRNAs induced by bacterial pathogens. For example, Toll-like-receptor (TLR)4 activation of primary human monocytes stimulated with LPS led to differential expression of a comprehensive set of lncRNAs. Two hundred and twenty-one out of nine hundred and eighty nine lncRNAs showed LPS-induced differences in expression levels (Ilott et al., 2014). Interestingly, differentially expressed lncRNAs were closely located to differentially expressed inflammatory genes. Another group investigated publicly available microarray data of LPS stimulated mouse macrophages and identified several lncRNAs to be differentially regulated upon stimulation. They found a correlation in the expression of many lncRNAs and neighboring protein-coding genes, for example NFκB2 or Rel, both involved in NFκB signaling (Mao et al., 2015).

In THP-1 macrophages infected with *C*. *concisus* 333 long intergenic RNAs (lincRNAs, a subclass of lncRNAs) were differentially expressed compared to uninfected cells (Kaakoush et al., 2015). Among these, eight were exclusively expressed in infected cells. The functions of these eight lincRNAs are unknown but their adjacent protein coding genes were shown to be involved in the regulation of immune responses. For example, in *C. concisus* infected cells the protein coding gene TNFAIP3 (which was upregulated 25.7-fold by the infection) adjacent to regulated lncRNAs was shown to inhibit TNF- and IL1-induced NFκB gene expression (Song et al., 1996). It was shown that TNFAIP3 is induced by TNF and IL-1 and might therefore be involved in the regulation of NFκB dependent genes in diverse bacterial infections. Consequently, the lncRNA adjacent to TNFAIP3 induced by *C. concisus* might be a NFκB regulator influenced by several pathogens known to affect NFκB signaling. In accordance with this, Ma et al. recently demonstrated that there is a lncRNA (lincRNA-Tnfaip3) located at mouse chromosome 10 proximal to TNFAIP3 which acts as an early response gene controlled by NFκB signaling in LPS stimulated mouse macrophages (Ma et al., 2016). LincRNA-Tnfaip3 seems to assemble a NFκB/Hmgb1/lincRNA-Tnfaip3-complex in response to LPS which leads to epigenetic chromatin remodeling and transactivation of inflammatory genes in mouse macrophages.

Speculating on lncRNA function based on their locus and function of neighboring protein coding genes might therefore be useful to predict potential lncRNA mechanisms and identify possible targets to discover gene regulatory functions during infection. However, only a fraction of reported lncRNAs involved in the immune response toward bacterial infection were validated in terms of mechanisms and mode of action. Directed approaches as described below and suitable knock-out models are needed to predict and functionally explore suchlike lncRNAs in host organisms infected with bacterial pathogens.

## Functional and mechanistic studies

A wealth of research projects have been carried out to elucidate the mechanisms how small ncRNAs, especially miRNAs, interact with their target molecules and post-transcriptionally inhibit gene function during bacterial infections. However, in case of lncRNAs the current knowledge is restricted to a handful of studies that investigated the functional role of certain lncRNAs during pathogen challenge or stimulation with pathogen associated molecular patterns (PAMPs). Some lncRNAs mediate their function through similar mechanisms e.g., promoting changes in chromatin methylation states or interacting with heterogenous ribonucleoproteins (hnRNPs). For other lncRNAs the function could be associated to a specific pathway, however, the molecular mode of action by which the function is mediated remains to be explored. A summary of lncRNAs that were validated in terms of function and/or mode of action is listed in Table 1.

**Table 1:** **Validated functions and mechanisms of lncRNAs expressed in host cells during bacterial infection or stimulation with bacterial compounds**.

**lncRNA**	**Host organism**	**Pathogen/microbial component**	**Function**	**Mechanism**	**References**
LincRNA-Tnfaip3	Mouse macrophages	LPS	Activation and repression of distinct classes of inflammatory genes	Assembles a NFkB /Hmgb1/lincRNA-Tnfaip3 complex which modulates Hmgb1-associated histone modifications	Ma et al., 2016
lncRNA- CD244	CD8+ T cells	*M. tuberculosis*	Inhibition of TNF-α and INF-γ expression	Interaction with PCR2	Wang et al., 2015
Unnamed lncRNAs	THP-1 cells	LPS	Regulates TNF-α expression	Interaction with PCR2 subunit EZH2, SUZ12 and LRRFIP1	Shi et al., 2014
lncRNA-IL7R	THP-1	LPS, TLR2-ligand	Reduces the expression of LPS induced inflammatory mediators such as E-selectin and VCAM-1	Increases trimethylation of H3K27	Cui et al., 2014
NeST	Mice	*Salmonella* (and Theiler's virus)	INF-γ upregulation	Interaction with WDR5	Gomez et al., 2013
lincRNA- Cox2	Mouse macrophages	LPS, TLR2-ligand, *L. monocytogenes*	Regulates the expression of immune related genes	Interaction with hnRNP	Carpenter et al., 2013
	Mouse bone marrow derived dendritic cells	LPS			Guttman et al., 2009
THRIL	THP-1	TLR2-ligand	Regulates TNF-α expression	Interaction with hnRNP	Li et al., 2014
lincRNA- EPS	Macrophages and dendritic cells	*L. monocytogenes*, TLR2-, TLR3-, TLR4-ligands	Controls immune related genes	Interaction with hnRNP	Atianand et al., 2016
lncRNA AS-IL1α	Mouse macrophages	*L. monocytogenes*, TLR ligands	Enhances IL1α expression	recruits RNA polymerase II to the IL1-α promotor	Chan et al., 2015
MEG3	THP-1 cells	*M. bovis* BCG	autophagy	Unknown	Pawar et al., 2016a

### lncRNA-mediated modulation of chromatin methylation states

Several lncRNAs were shown to control transcription of mRNAs by turning the methylation state of their target genes to an active or suppressive condition.

For example, the Polycomp Repressive Complex 2 (PCR2) exhibits methyltransferase activity through its subunit EZH2 and leads to trimethylation of a lysine residue at position 27 in the protein histone H3 (H3K27me3). H3K27me3 is a marker for transcriptional repression. PCR2 consists of several subunits. Two of them, mentioned EZH2 (exhibits methyltransferase activity) and SUZ12 (a zinc-finger protein) have been reported to interact with lncRNAs to inhibit expression of target genes by repressing chromatin at the respective promotors (Khalil et al., 2009).

TNF-α seems to be a prominent target regulated by this mechanism during bacterial infections. It was shown that the lncRNA-CD244 which is upregulated by the T-cell inhibitory molecule CD244 in *M. tuberculosis* infection acts as an epigenetic inhibitor of TNF-α and INF-γ expression. The authors were able to show that lncRNA-CD244 directly interacts with the PCR2 subunit EZH2 which leads to trimethylation of H3K27 and a more repressive chromatin state at the INF-γ or TNF-α loci (Wang et al., 2015). A similar mechanism in terms of lncRNA-mediated control of TNF expression has been reported through bidirectional lncRNAs upstream of the TNF gene locus (Shi et al., 2014). The authors were able to show that these unnamed lncRNAs bind LRRFIP1 (a repressor of TNF) and chromatin. Furthermore, the data suggests a scaffolding function for these lncRNAs by which the 2 subunits of the PCR2 complex (EZH2 and SUZ12) and LRRFIP1 are assembled to the TNF region to maintain a repressed chromatin state at the TNF promotor forming an inhibitory complex.

TNF-α plays a major role in the host response to control *M. tuberculosis* infection. To promote infection and ensure persistence and replication in the host cell, *M. tuberculosis* is known to successfully inhibit TNF-α production via diverse mechanisms including modulation of the miRNA host response (Reed et al., 2004; Kurtz et al., 2006; Singh et al., 2013). Depending on the cellular context, TNF can either induce apoptosis or NFκB-mediated survival. Many bacterial pathogens such as *S*. Typhimurium or *Yersinia enterocolitica* are known to interfere with TNF signaling to promote pathogenicity (Rahman and McFadden, 2006). Modulation of TNF expression through lncRNAs is a new reported mechanism which might possibly be employed by other pathogens interfering with TNF levels to establish infection.

Another lncRNA affecting the trimethylation state of H3K27 is lnc-IL7R. It was highly induced in THP-1 cells as well as PBMCs when they were stimulated with TLR4- and TLR2- but not with TLR3-ligands (Cui et al., 2014). By increasing trimethylation of H3K27 at the proximal promotor, lnc-IL7R was able to reduce the expression of LPS-induced inflammatory mediators such as E-selectin and VCAM-1. However, the mechanism by which the trimethylation state of H327k was affected could not be elucidated since the authors found no interaction between lnc-IL7R and members of the PCR2 complex or other protein binding partners of lnc-IL7R.

In contrast to generating a repressive chromatin state, the lncRNA NeST has been shown to turn chromatin to an active state by binding WDR5 in the Histone 3 Lysine 4 methyltransferase complex (H3K4). The trimethylation of H3K4 establishes an active chromatin state at the INF-γ locus leading to INF-γ accumulation. Initially the lncRNA NeST was linked to Theiler virus susceptibility. Gomez et al. found out that it controls the pathogenesis of another pathogen. They demonstrated that mice expressing NeST RNA show increased susceptibility to Theiler but decreased *Salmonella* pathogenicity (Gomez et al., 2013). Binding of the adapter protein WDR5 by lncRNAs to generate an active chromatin state at H3K4 has been shown for other lncRNAs (e.g., HOTTIP) and might be demonstrated for further lncRNAs in the future.

### The role of NFκB

As described above, many lncRNAs were reported to interfere with NFκB signaling (Ilott et al., 2014; Ma et al., 2016). NFκB proteins are a family of five structurally related transcription factors controlling the expression of inflammatory molecules to counteract bacterial infection. The process of NFκB activation is mediated via recognition of pathogen-associated-molecular patterns (PAMPS) on the outer side of the microbe through respective pattern-recognition receptors such as TLRs found on the membrane and in the cytoplasm of host cells. To overcome immune response, bacteria have developed mechanism to modulate NFκB signaling (Johannessen et al., 2013).

Interestingly, the expression of several lncRNAs has been linked to TLR-dependent NFκB activation. This suggests that lncRNAs are involved in NFκB signaling regulating the transcription of not only cytokines during host response but also regulating several aspects of innate and adaptive immune response such as differentiation, proliferation and survival. An example is lincRNA-Cox2. It has been first reported to be upregulated in mouse bone-marrow-derived dendritic cells after TLR4 stimulation while it was only slightly increased upon TLR3 stimulation. It is located 51 kb upstream of the Cox2 coding gene and induced directly via NκFκB (Guttman et al., 2009). Similarly, in another study, lincRNA-Cox2 was among the most highly induced lncRNA candidates in mouse macrophages when stimulated with the TLR2 ligand Pam3CSK4 but also induced by TLR4- and TLR7/8-stimulation as well as *L. monocytogenes* infection (Carpenter et al., 2013). Although it was shown to form a complex with hnRNP A/B and A2/B1, no direct target of lincRNA-Cox 2 was identified. However, the authors were able to show that lincRNA-Cox2 both positively and negatively regulated the expression of immune related genes.

The interaction with hnRNPs to influence TLR/NFκB signaling has also been demonstrated for other lncRNAs. The lncRNA THRIL (TNF-α and hnRNPL related immunoregulatory LincRNA) has been found to be dysregulated in human THP-1 macrophages stimulated with TLR2 ligand. The authors found that THRIL regulates TNF-α expression by interacting with hnRNP-L (Li et al., 2014). THRIL and hnRNP-L possibly form a complex that stimulates TNF-α transcription by binding to the TNF-α promotor. Furthermore, the expression of many other NFκB-dependent pro-inflammatory cytokines were influenced by THRIL, demonstrated through THRIL knockdown. Atianand et al. identified another lncRNA (lincRNA-EPS) that controls NFκB dependent immune related genes in TLR-stimulated as well as *L. monocytogenes* infected macrophages and dendritic cells by binding hnRNPs (Atianand et al., 2016). Besides lincRNA-Cox2 and lincRNA-EPS another lncRNA called AS-IL1α has been shown to be induced after *L. monocytogenes* infection of mouse macrophages as well as stimulation with a range of TLR ligands (Pam3CSK4-TLR2, LPS-TLR4, polyinosinic-polycytidylic acid-TLR3) in a NFκB dependent fashion. AS-IL1α is partially complementary to IL1α and has been shown to enhance IL1α expression by recruiting RNA pol II to the IL1α promotor (Chan et al., 2015).

The question if lncRNA-interference with NFκB and other immune related pathways favors the host or the pathogen needs to be investigated under defined conditions. The lncRNAs expressed in response to TLR stimulation might belong to a core response to fine tune immune function as described for microRNAs (Siddle et al., 2015). If there are specific lncRNA candidates manipulated by bacterial effector proteins or individual virulence factors needs to be in the focus of further investigations. Interestingly, viable but not heat killed *M. bovis* BCG were able to downregulate the lncRNA MEG3 in macrophages indicating a participation of effectors derived from viable bacteria influencing lncRNA expression (Pawar et al., 2016a).

### Interaction with microRNAs

As described in the introduction, there is evolving evidence that lncRNAs and miRNAs influence each other through diverse mechanisms (Yoon et al., 2014). However, until today there is no data confirming this crosstalk in host cells during bacterial infection.

Nevertheless, our group recently identified a participation of the lncRNA MEG3 in the process of autophagy in macrophages infected with *M. bovis* BCG based on the hypothesis that lncRNAs and miRNAs directly interact. Pathway analysis and subsequent follow-up experiments revealed MEG3 to exhibit a regulatory function in autophagy (Pawar et al., 2016a). Although, the exact mechanism and miRNA-interaction partner remains to be elucidated in this case, we presume that diverse functions of lncRNAs involved in bacterial infections are mediated through their interaction with miRNAs.

## Conclusion/outlook

According to the reviewed literature, lncRNAs seem to be important epigenetic regulators of the mammalian immune response toward bacterial infection. However, lncRNA research is still in its infancy compared to research on miRNAs, especially regarding their role in bacterial diseases. Not only based on their length but also because of heterogeneous mechanisms and molecular modes of action lncRNAs completely differ from miRNA and are believed to prompt substantial clues in regulation of host response to bacterial infections.

Since miRNAs were first described in 1993, the use of miRNAs as a diagnostic tool but also their therapeutic application for various types of diseases are a major focus of investigation and initial miRNA-based therapeutics are currently examined in clinical trials (Li and Rana, 2014). So far, it appears that the use of lncRNAs as a diagnostic marker and therapeutic agents might be more prosperous since lncRNAs seem to be tissue- and target-specific in contrast to miRNAs which are master-regulators in several tissues and often target multiple mRNAs. This could make lncRNAs more specific biomarkers but also promising therapeutics for a certain disease due to minimized off-target effects. However, a lncRNA exhibiting exclusively pathogenic or beneficial potential during infection has yet not been described. Furthermore, it is still unknown if there are specific lncRNAs expressed in response to a certain pathogen or if lncRNAs are mainly involved in basic cellular immune responses to different stress stimuli. In case of miRNAs it was shown that a core temporal miRNA expression pattern is induced in response to infection, shared across different bacterial species. In addition, pathogen-specific miRNAs were identified reflecting the mechanism by which certain pathogens interfere with the host response to infection (Siddle et al., 2015). Studies comparing the lncRNA expression in response to different pathogens possessing different virulence mechanisms are missing. However, it was shown that two strains of *M. tuberculosis* that differ in virulence induced distinct lncRNA expression profiles (Yang X. et al., 2016). Regarding the therapeutic application, lncRNAs are interesting candidates for host-directed therapies which is a new evolving concept to treat bacterial infections such as tuberculosis. Targeting the host rather than the pathogen prevents development of antibiotic resistance and supports immune defense mechanisms during infection (Zumla et al., 2016). Influencing the expression of lncRNAs that augment cellular antimicrobial mechanisms such as autophagy or directly reducing inflammation is therefore a promising approach, especially to treat multi-drug-resistant infections.

Finally, there is still a long journey ahead to identify and elucidate cellular functions and mechanisms of lncRNAs regulated by bacterial pathogens.

## Author contributions

JZ and SS literature research, wrote manuscript; RE: wrote manuscript.

### Conflict of interest statement

The authors declare that the research was conducted in the absence of any commercial or financial relationships that could be construed as a potential conflict of interest.
